# Metabolomic Profiling of Plasma from Melioidosis Patients Using UHPLC-QTOF MS Reveals Novel Biomarkers for Diagnosis

**DOI:** 10.3390/ijms17030307

**Published:** 2016-02-27

**Authors:** Susanna K. P. Lau, Kim-Chung Lee, George C. S. Lo, Vanessa S. Y. Ding, Wang-Ngai Chow, Tony Y. H. Ke, Shirly O. T. Curreem, Kelvin K. W. To, Deborah T. Y. Ho, Siddharth Sridhar, Sally C. Y. Wong, Jasper F. W. Chan, Ivan F. N. Hung, Kong-Hung Sze, Ching-Wan Lam, Kwok-Yung Yuen, Patrick C. Y. Woo

**Affiliations:** 1 State Key Laboratory of Emerging Infectious Diseases, The University of Hong Kong, Pokfulam, Hong Kong, China; kelvinto@hkucc.hku.hk (K.K.W.T.); kyyuen@hkucc.hku.hk (K.-Y.Y.); 2 Research Centre of Infection and Immunology, The University of Hong Kong, Pokfulam, Hong Kong, China; ivanhung@hku.hk; 3 Carol Yu Centre for Infection, The University of Hong Kong, Pokfulam, Hong Kong, China; 4 Department of Microbiology, The University of Hong Kong, Pokfulam, Hong Kong, China; leekimchung1983@gmail.com (K.-C.L.); gcslo@connect.hku.hk (G.C.S.L.); sydin1@hku.hk (V.S.Y.D.); cwn5810@gmail.com (W.-N.C.); kyh416@hotmail.com (T.Y.H.K.); shirly.curreem@gmail.com (S.O.T.C.); tipyin@yahoo.com.hk (D.T.Y.H.); sid8998@gmail.com (S.S.); wcy288@ha.org.hk (S.C.Y.W.); jfwchan@hku.hk (J.F.W.C.); khsze@hku.hk (K.-H.S.); 5 Department of Medicine, The University of Hong Kong, Pokfulam, Hong Kong, China; 6 Department of Pathology, The University of Hong Kong, Pokfulam, Hong Kong, China; ching-wanlam@pathology.hku.hk

**Keywords:** *Burkholderia pseudomallei*, melioidosis, plasma, metabolomics, biomarkers

## Abstract

To identify potential biomarkers for improving diagnosis of melioidosis, we compared plasma metabolome profiles of melioidosis patients compared to patients with other bacteremia and controls without active infection, using ultra-high-performance liquid chromatography-electrospray ionization-quadruple time-of-flight mass spectrometry. Principal component analysis (PCA) showed that the metabolomic profiles of melioidosis patients are distinguishable from bacteremia patients and controls. Using multivariate and univariate analysis, 12 significant metabolites from four lipid classes, acylcarnitine (*n* = 6), lysophosphatidylethanolamine (LysoPE) (*n* = 3), sphingomyelins (SM) (*n* = 2) and phosphatidylcholine (PC) (*n* = 1), with significantly higher levels in melioidosis patients than bacteremia patients and controls, were identified. Ten of the 12 metabolites showed area-under-receiver operating characteristic curve (AUC) >0.80 when compared both between melioidosis and bacteremia patients, and between melioidosis patients and controls. SM(d18:2/16:0) possessed the largest AUC when compared, both between melioidosis and bacteremia patients (AUC 0.998, sensitivity 100% and specificity 91.7%), and between melioidosis patients and controls (AUC 1.000, sensitivity 96.7% and specificity 100%). Our results indicate that metabolome profiling might serve as a promising approach for diagnosis of melioidosis using patient plasma, with SM(d18:2/16:0) representing a potential biomarker. Since the 12 metabolites were related to various pathways for energy and lipid metabolism, further studies may reveal their possible role in the pathogenesis and host response in melioidosis.

## 1. Introduction

Melioidosis is a disease caused by the highly pathogenic gram-negative bacterium, *Burkholderia pseudomallei* (*B. pseudomallei*). The disease is often serious and potentially fatal, most commonly manifested as severe community-acquired pneumonia and sepsis. Although the disease is mainly endemic in Southeast Asia and Northern Australia, melioidosis has been increasingly reported in areas of the Asia-Pacific region, including India [1,2], Mauritius [3], South, Central and North America [4,5,6], and West and East Africa [7,8], which may suggest an expanding geographical distribution. *B. pseudomallei* is known to be a natural saprophyte, and therefore melioidosis is believed to be acquired through contact with contaminated soil and water in the environment [9,10]. Illness can be presented as an acute, subacute, or chronic process, with an incubation period of up to 26 years [11]. The disease manifestations can range from subclinical infection, localized abscesses, and pneumonia to fulminant sepsis, leading to a mortality rate of up to 19% [12]. Besides human, melioidosis also affects various animals in endemic areas [10,13]. Treatment of melioidosis is often difficult, as *B. pseudomallei* is usually resistant to multiple antibiotics and prolonged antibiotics are required to prevent relapse [14,15]. Moreover, diagnostic and therapeutic resources in endemic areas are often limited, which have hindered efforts to improve treatment outcomes.

Diagnosis of melioidosis can be difficult because of several reasons. First, *B. pseudomallei* may not be readily isolated from clinical specimens. Second, even if it is successfully isolated, commercial bacterial identification systems often cannot differentiate *B. pseudomallei* from closely related species such as *Burkholderia thailandensis* and members of *Burkholderia cepacia* complex [16]. Therefore, new molecular techniques are often required for more accurate species identification [14,17,18,19,20,21,22,23]. Despite these new technologies, the diagnostic problems associated with culture-negative cases remain unresolved. Although different serological tests have been developed to help diagnose culture-negative melioidosis, their clinical usefulness is limited by the low sensitivities and specificities [24,25]. The availability of alternative techniques for improved diagnosis of melioidosis is thus eagerly awaited, and such techniques should be able to differentiate between melioidosis and infections caused by common Gram-negative bacteria including the closely related *Burkholderia* species.

Metabolomics is a new research platform for systematic studies of small molecules of a specific system such as a cell, tissue or an organism. The metabolic profiles between such systems can then be compared, thus allowing the identification of specific metabolite markers. The technique has been used to characterize various infectious diseases or pathogens [26,27,28,29,30,31,32,33,34,35,36,37,38]. By exploring the metabolomes of culture supernatant, we have identified specific biomarkers that are produced by a unique thiamine degradation pathway in *B. pseudomallei* [32]. We have also recently reported the use of metabolomics to identify novel biomarkers in plasma of tuberculosis patients, which may be useful for diagnosis [37]. Despite being an important pathogen, no studies have reported the use of metabolomics to explore specific biomarkers in plasma of melioidosis patients.

We hypothesize that there are specific biomarkers that may be detected in plasma of melioidosis patients. To identify potential biomarkers for the non-invasive diagnosis of melioidosis, we applied the metabolomics technology for metabolite profiling of plasma samples from melioidosis patients, using ultra-high-performance liquid chromatography-electrospray ionization-quadrupole time-of-flight mass spectrometry (UHPLC-ESI-QTOFMS). Multi- and univariate statistical analyses of the metabolome data were used to identify specific metabolites that are present in significantly higher levels in plasma of melioidosis patients than in plasma of patients with other bacteremia or controls without infections. The diagnostic performances of the identified biomarkers were evaluated using receiver operating characteristic curve (ROC) analysis. In this pilot study, untargeted metabolomics on plasma sample were conducted with the aim to explore potential diagnostic biomarkers and biological pathways involved in host–*B. pseudomallei* interaction.

## 2. Results

### 2.1. Metabolomic Profiling of Plasma Samples from Melioidosis Patients, Patients with Other Bacteremia and Controls

The metabolomes of 76 plasma samples from the three groups (22 samples from newly-diagnosed melioidosis patients, 24 samples from patients with other bacteremia and 30 samples from controls without active infection) were compared using UHPLC-QTOFMS [38]. The metabolites could be separated well using the UPLC-MS method with sub-micron particle size, 1.7 µm packing. The base peak chromatographic profiles showed stable retention time for all peaks without observable drift, supporting the stability and reliability of the accurate-mass QTOF system and metabolomic profiling data.

### 2.2. Omics-Based Statistical and Bioinformatic Analysis for Identification of Biomarkers

**Figure 1:**
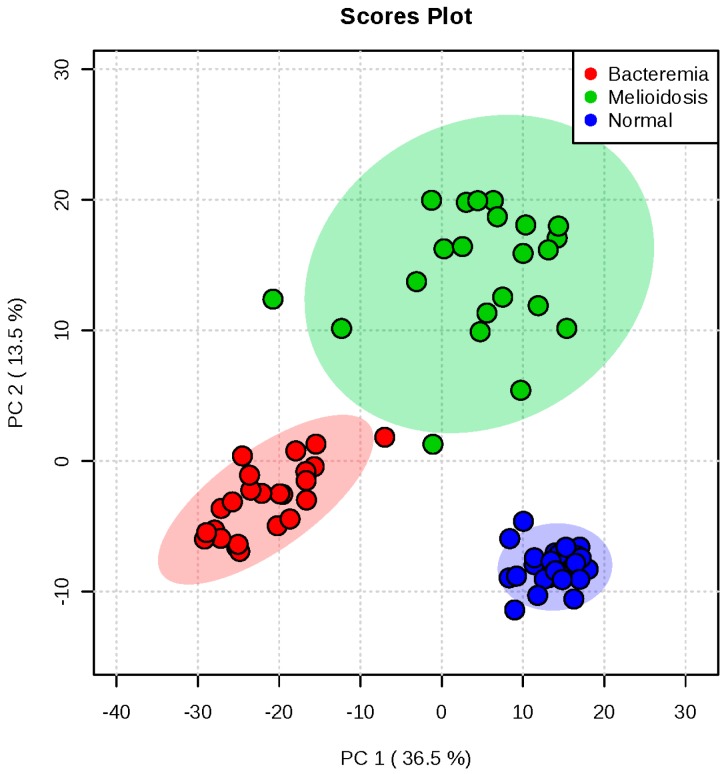
Principal component analysis (PCA) score plot in positive mode based on human plasma of 22 melioidosis, 24 bacteremia and 30 controls without active infection. The PCA score plots showed that samples from melioidosis patients, bacteremia patients and controls without active infection were clustered separately.

A total of 2424 molecular features were obtained by XCMS package [39] and subjected to MetaboAnalyst 3.0 software [40] for statistical analysis. For multivariate analysis, principal component analysis (PCA) revealed that the three groups were clustered separately, with 50.0% of the total variance among the three groups represented by the first two principal components (PCs), where principal component 1 (PC1) and PC2 explained 36.5% and 13.5% of the variance, respectively (Figure 1). In particular, the melioidosis group could be distinguished from the bacteremia and control groups based on the first two PCs, with clear separation along PC2 dimension.

**Figure 2:**
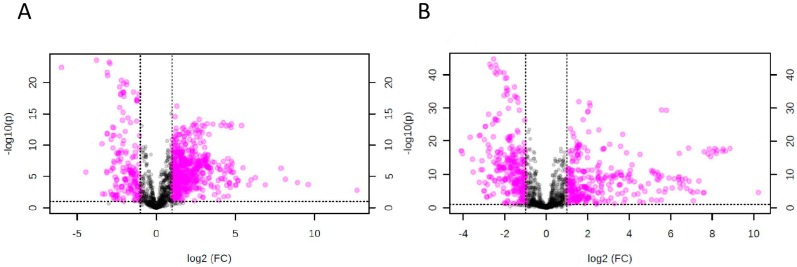
Volcano Plot, using fold-change (FC) >1.5 and *p*-values cut-off <0.05 using Student’s *t*-test. The statistical analyses were performed for comparison between melioidosis and bacteremia patients in (**A**) positive mode as well as melioidosis patients and controls without active infection in (**B**) positive mode.

Univariate analysis using one-way analysis of variance (ANOVA) identified 764 statistically significant features with variable importance in the projection (VIP) score >1 and *p* < 0.05 when compared both between melioidosis patients and patients with other bacteremia, and between melioidosis patients and controls. Further volcano plot analysis revealed 131 significant features with fold-change (FC) >1.5 and *p* < 0.05 by Student’s t-test when compared both between melioidosis patients and patients with other bacteremia (Figure 2A), and between melioidosis patients and controls (Figure 2B), which were subjected to univariate ROC analysis.

**Figure 3:**
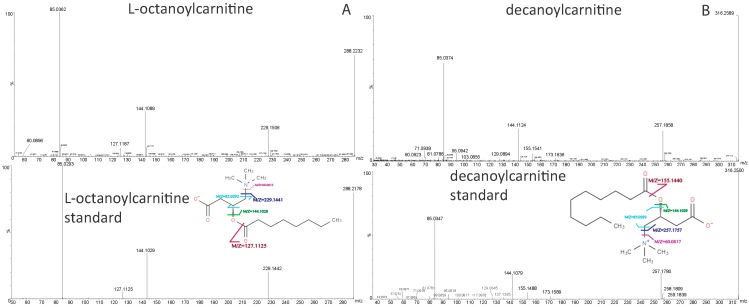

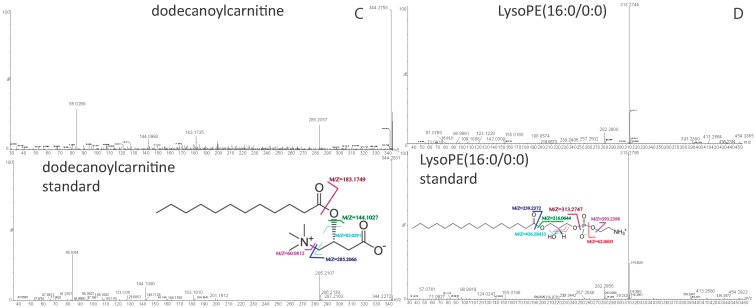

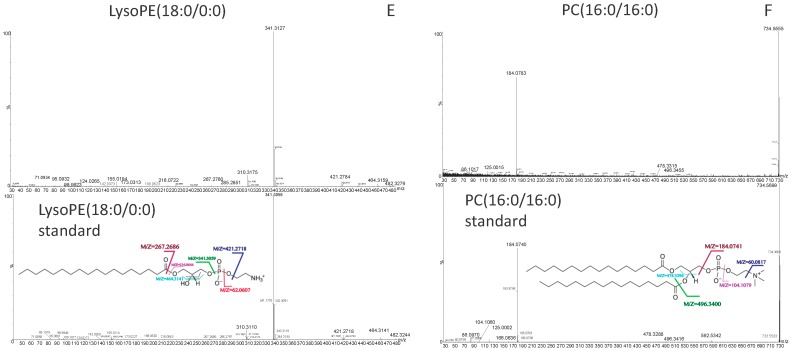

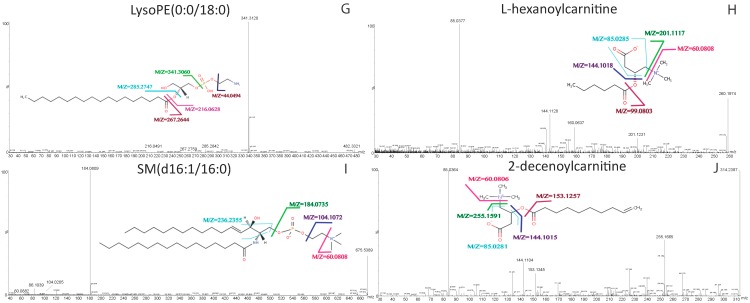

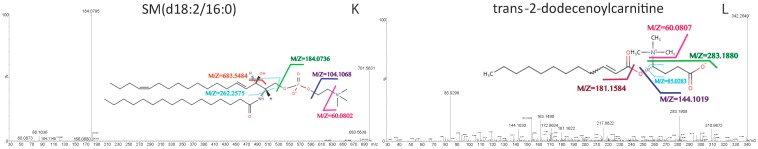
MS/MS mass spectra and predicted structures with expected fragmentation profiles of the 12 biomarkers in melioidosis patient plasma: (**A**) l-octanoylcarnitine; (**B**) decanoylcarnitine; (**C**) dodecanoylcarnitine; (**D**) lysophosphatidylethanolamine (LysoPE)(16:0/0:0); (**E**) LysoPE(18:0/0:0); (**F**) phosphatidylcholine PC(16:0/16:0); (**G**) LysoPE(0:0/18:0) (**H**) l-hexanoylcarnitine; (**I**) sphingomyelins SM(d16:1/16:0); (**J**) 2-decenoylcarnitine; (**K**) SM(d18:2/16:0); and (**L**) *trans*-2-dodecenoylcarnitine with or without comparison to commercially available standards.

A total of 12 metabolites with area-under-receiver operating characteristic curve (AUC) >0.80 when compared between melioidosis patients and patients with other bacteremia, or between melioidosis patients and controls, were identified. They were identified as metabolites belonging to four lipid classes, acylcarnitine (six metabolites), lysophosphatidylethanolamine (LysoPE) (three metabolites), sphingomyelins (SM) (two metabolites) and phosphatidylcholine (PC) (one metabolite), using LC-MS/MS analyses by elution order, MS/MS fragmentation and predicted molecular formula (Table 1 and Table 2, Figure 3). The identification and assignment of lipid classes were based on the fingerprint fragment and specific neutral losses. Specifically, the presence of m/z 60 (trimethylamine ion, C_3_H_10_N^+^) and m/z 85 (C_4_H_5_O_2_^+^) for acylcarnitine, m/z 44 (C_2_H_5_N^+^) and neutral loss of phosphorylethanolamine for LysoPE, m/z 184 (Phosphocholine ion, C_5_H_15_NO_4_P^+^) and neutral loss of water for SM, and m/z 60 (trimethylamine ion, C_3_H_10_N^+^) and m/z 184 (phosphocholine ion, C_5_H_15_NO_4_P^+^) for PC, were key features employed for their identification. In addition, the identities of six biomarkers, including decanoylcarnitine, decanoylcarnitine, and l-octanoylcarnitine LysoPE(16:0/0:0), LysoPE(18:0/0:0) and PC(16:0/16:0), were confirmed by matching the retention time (RT) and MS/MS fragmentation patterns of authentic chemical standards, where available. Chemical standards for the other six metabolites were not available for comparison. The details of the fragments in each MS/MS spectrum for each identified metabolite are shown in Figure 3. The 12 metabolites were related to various pathways for energy and lipid metabolism, including tricarboxylic acid (TCA) cycle, fatty acid β-oxidation, fatty acid de novo synthesis, linoleic acid, α-linoleic acid, arachidonic acid, phospholipid and sphingolipid metabolism.

### 2.3. Diagnostic Performance of Metabolites

**Figure 4:**
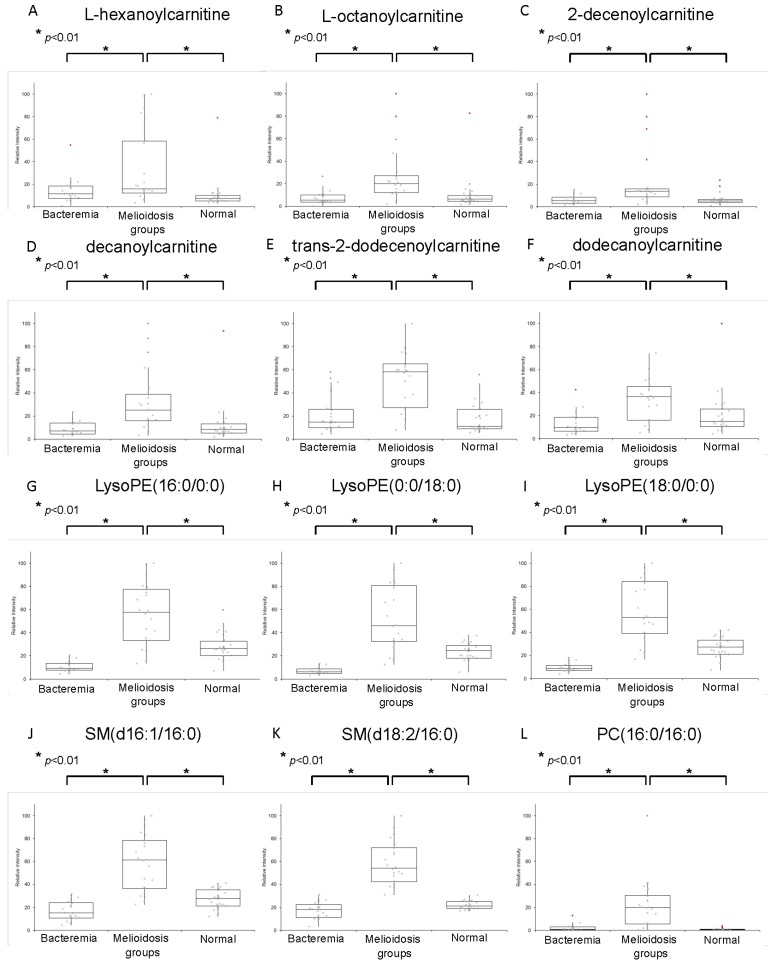
Box-and-whiskers plots representing relative abundance of: (**A**) l-hexanoylcarnitine; (**B**) l-octanoylcarnitine; (**C**) 2-decenoylcarnitine; (**D**) decanoylcarnitine; (**E**) *trans*-2-dodecenoylcarnitine; (**F**) dodecanoylcarnitine; (**G**) LysoPE(16:0/0:0); (**H**) LysoPE(0:0/18:0); (**I**) LysoPE(18:0/0:0); (**J**) SM(d16:1/16:0); (**K**) SM(d18:2/16:0); and (**L**) PC(16:0/16:0) in plasma of melioidosis patients, bacteremia patients and controls without active infections. The relative abundance of each metabolite in plasma of melioidosis patients was significantly higher than the other two groups using Student’s *t*-test (*p*-value < 0.01).

**Figure 5:**
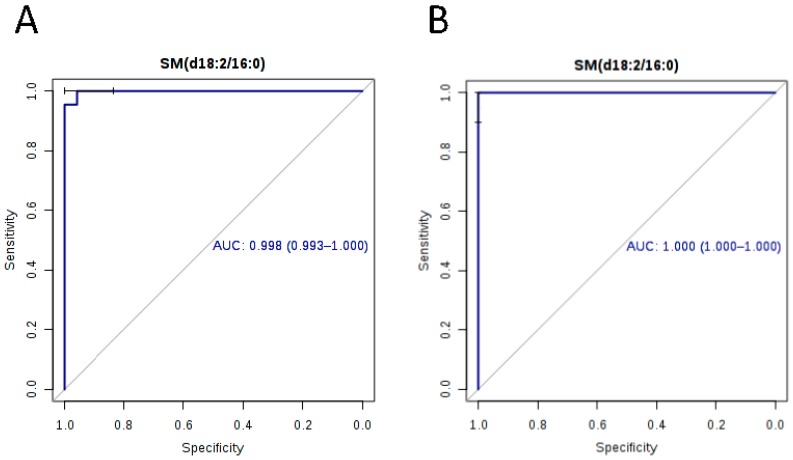
Receiver operating characteristic (ROC) curves for SM(d18:2/16:0) with Area Under the Curves (AUC) with 95% confidence interval, when compared (**A**) between melioidosis and other bacteremia patients; and (**B**) between melioidosis patients and controls without active infection.

The AUC, sensitivity and specificity for ROC curves calculated for the 12 metabolites at optimal cutoffs are summarized in Table 2. Box-whisker plots revealed that they all exhibited significantly higher levels in plasma samples of melioidosis patients than in samples of patients with other bacteremia and controls (*p* < 0.01 by Student’s *t*-test) (Figure 4). Among the 12 biomarkers, 10 showed AUC > 0.80 when compared both between melioidosis and bacteremia patients, and between melioidosis patients and controls. SM(d18:2/16:0) possessed the largest AUC when compared both between melioidosis patients and patients with other bacteremia (AUC 0.998, sensitivity 100% and specificity 91.7%), and between melioidosis patients and controls (AUC 1.000, sensitivity 96.7% and specificity 100%) (Figure 5).

## 3. Discussion

Using metabolomics approach, we identified 12 novel biomarkers in plasma of melioidosis patients with significantly higher levels than in plasma of patients with other bacteremia and controls without active infection. In this study, samples from patients with other bacteremia were included because these patients may present with sepsis mimicking melioidosis where *B. pseudomallei* bacteremia can occur. Therefore, the present biomarkers may help differentiate melioidosis from bacteremia caused by common bacterial species such as *Escherichia coli*. In particular, SM(d18:2/16:0) represents the most promising biomarker for melioidosis, with AUC of 0.998, sensitivity of 100.0% and specificity of 91.7% when compared to other bacteremia, and AUC of 1.000, sensitivity of 96.7% and specificity of 100.0% when compared to controls without active infection. However, the present study is limited by the small number of patients with melioidosis included, which is partly due to clinical difficulties in the diagnosis of melioidosis and our relatively low disease prevalence when compared to other Southeast Asian countries such as Thailand. Further studies with inclusion of more cases from endemic regions are required to validate the diagnostic potential of the present biomarkers.

The high plasma concentrations of six medium- to long-chain (C13 to C19) acylcarnitines in melioidosis patients may reflect changes in fatty acid (FA) β-oxidation during infection. Acylcarnitines are synthesized from acyl-CoAs with transfer of hydroxyl group of carnitine, and are transported from the intermembraneous space into the mitochondrial matrix for FA β-oxidation [41]. In mice infected with *B. pseudomallei*, transcriptomics studies have revealed changes in transcript levels of various enzymes involved in FA β-oxidation [42]. Interestingly, higher levels of medium-chain (C5 to C10) acylcarnitines have also been found in plasma of patients with systemic inflammatory response syndrome (SIRS) due to severe sepsis/septic shock than those with SIRS due to non-infective causes [43]. Since accumulation of medium-chain acyl-CoAs in the mitochondria is toxic for the cell, the synthesis of acylcarnitines may have a protective effect during infection [43]. Further studies may help elucidate the role of these medium- to long-chain acylcarnitines in the host response to melioidosis.

The high levels of three LysoPE, LysoPE (16:0/0:0), LysoPE (0:0/18:0) and LysoPE (18:0/0:0), in plasma of melioidosis patients may be the result of changes in phospholipid metabolism or cellular damage from systemic infection. LysoPE, a constituent of cell membranes, is derived from the hydrolysis of PE, which is catalyzed by phospholipase A2 (PLA2) [44]. It has been shown that LysoPEs can stimulate invariant natural killer T cell activation through self-antigenicity, suggesting a possible role in innate immunity during infection [45]. In addition, LysoPE was shown to enhance the ingestion activity of macrophage on IgG-coated target cells via Fc receptors [46]. It remains to be determined if LysoPE may be involved in the innate immunity against *B. pseudomallei*. Our findings may also be in line with previous observations of higher plasma levels of type II PLA2 in sepsis patients, which significantly correlated with TNF-α, IL-6 and IL-8 levels [47]. On the other hand, elevated plasma levels of LysoPE (16:0/0:0) have been observed in rats with induced-liver injuries as a result of massive destruction of cell membranes [48,49]. Therefore, it is also possible that the high LysoPE levels may be the consequence of severe organ damage during systemic melioidosis.

The high levels of SM(d16:1/16:0) and SM(d18:2/16:0) may reflect changes in sphingolipid metabolism during melioidosis. SMs, structural components of cell membranes, are synthesized by the transfer of PC to ceramides by sphingomyelin synthase and degraded back to ceramides by sphingomyelinase [50]. While these SMs are likely produced by the host, it is interesting to note that the genome of *B. pseudomallei* also possessed genes homologous to hemolytic phospholipase C (PlcH) of *Pseudomonas aeruginosa* with sphingomyelin synthase and sphingomyelinase activity [50,51,52]. Further studies are required to better understand the possible role of sphingomyelin metabolism in the pathogenesis of melioidosis.

The PC(16:0/16:0) detected in plasma of melioidosis patients may be the result of innate immune response in the lungs. PC(16:0/16:0) or dipalmitoylphosphatidylcholine (DPCC) is a major component of pulmonary surfactant [53,54]. Melioidosis is believed to be acquired through inhalation of contaminated aerosals. *B. pseudomallei* has been found to induce pro-inflammatory cytokines from macrophages and alveolar type II pneumocytes (ATII) cells [55], the latter being responsible for secretion of surfactants. We speculate that the production of PC(16:0/16:0) may be upregulated in ATII cells when in contact with *B. pseudomallei*, which may be secreted in plasma leading to the elevated PC(16:0/16:0) levels. However, comparison with plasma of patients with other causes of pneumonia would be important to determine if PC(16:0/16:0) is specific to melioidosis or may represent a general biomarker for pneumonia.

## 4. Materials and Methods

### 4.1. Patient and Control Samples

Clinical samples were collected from patients hospitalized in Queen Mary Hospital, Hong Kong. A total of 22 plasma samples from five patients with newly-diagnosed melioidosis, 24 plasma samples from 24 patients with bacteremia caused by other bacterial species and 30 controls without active infections were included for UHPLC-QTOFMS analysis. Plasma samples from melioidosis patients were collected before commencement of antibiotic treatment. The diagnosis of melioidosis was made according to compatible clinical features, and with either isolation of *B. pseudomallei* from clinical samples and/or positive antibodies against *B. pseudomallei* as determined by enzyme-linked immunosorbent assay as described previously [14]. All five patients with melioidosis were immunocompromised with underlying diseases, including HIV, autoimmune vasculitis, diabetes mellitus, lymphoma and acute myeloid leukemia. *B. pseudomallei* was isolated from the blood cultures of three patients, while the other two cases were diagnosed by positive antibodies against *B. pseudomallei*. Plasma samples were collected at admission from patients with other bacteremia were used. Causative agents of other bacteremia included *Aeromonas caviae* (*n* = 1), *Bacteroides* (*n* = 1), *E. coli* (*n* = 17), *Klebisella pnuemoniae* (*n* = 1), *Prevotella* species (*n* = 1), *Proteus mirabilis* (*n* = 2), *Streptococcus mitis* (*n* = 1), and *Streptococcus pneumoniae* (*n* = 2) (two patients had two different bacterial isolates from the same blood culture). Controls included patients with no clinical evidence of active infection. This study has been approved by the Institutional Review Board, the University of Hong Kong/Hospital Authority of Hong Kong West Cluster under reference number UW 13-265.

### 4.2. Chemicals and Reagents

LC-MS grade water, methanol and acetonitrile were purchased from J.T. Baker (Center Valley, PA, USA). High-performance liquid chromatography (HPLC)-grade ethanol and acetone were purchased from Merck & Co. (Kenilworth, NJ, USA). Formic Acid was of American Chemical Society (ACS) reagent grade from Sigma-Aldrich (Saint Louis, MO, USA). Decanoylcarnitine, LysoPE(16:0/0:0), LysoPE(18:0/0:0) and PC(16:0/16:0) was purchased from Avanti Polar Lipid (Alabaster, AL, USA). Decanoylcarnitine and l-octanoylcarnitine were purchased from Sigma-Aldrich (Saint Louis, MO, USA).

### 4.3. Sample Preparation

Blood samples were collected in heparin bottles, transferred immediately to the laboratory, and centrifuged at 3000 rpm at 4 °C for 10 min to obtain the plasma fractions. For metabolomics analysis, 100 µL of plasma was thawed at 4 °C and plasma proteins were precipitated with 400 µL of methanol/ethanol/acetone mixture at a ratio of 1:1:1 (*v*/*v*/*v*). The sample extract was vigorously vortexed for 1 min, and centrifuged at 14,000 rpm at 4 °C for 10 min. The supernatant was collected for UHPLC-ESI-QTOFMS analysis. All specimens were immediately kept at −80 °C until analysis and stored within one week. The thawed specimens were analyzed within 48 h in a random manner to prevent the batch effect.

### 4.4. Untargeted Metabolomics Profiling of Patient Plasma Using UHPLC-ESI-QTOFMS

The metabolomic profiling of plasma supernatants was performed as describe previously with modifications [37], using Agilent 1290 Infinity UHPLC (Agilent Technologies, Waldbronn, Germany) coupled with Agilent 6540 UHD Accurate-Mass QTOF system (Agilent Technologies, Santa Clara, CA, USA) accompanied with a MassHunter Workstation software for QTOF (version B.03.01 for Data Acquisition, Agilent Technologies, Santa Clara, CA, USA). Waters Acquity UPLC BEH C18 column (2.1 × 100 mm, 1.7 µm) (Waters, Milford, MA, USA) was used for the separation with the injection volume of 5 µL. The column and autosampler temperature were maintained at 45 and 10 °C, respectively. The separation was performed at a flow rate of 0.4 mL/min under a gradient program in which mobile phase A was composed of LC-MS grade water containing 0.1% formic acid (*v*/*v*) and mobile phase B was composed of acetonitrile. The gradient program was applied as follows: *t* = 0 min, 5% B; *t* = 0.5 min, 5% B; *t* = 7 min, 48% B; *t* = 20 min, 78% B; *t* = 27 min, 80% B; *t* = 31 min, 99.5% B; *t* = 36.5 min, 99.5% B; *t* = 36.51 min, 5% B. The stop time was 40 min. The ESI mass spectra were acquired in both positive and negative ion modes using Agilent Jet Stream ESI source (Agilent Technologies, Santa, CA, USA) with capillary voltages at +3800 and −3500 V, respectively. Other source conditions were kept constant in all the experiments as follow: gas temperature was kept constant at 300 °C, drying gas (nitrogen) was set at the rate of 7 L/min, and the pressure of nebulizer gas (nitrogen) was 40 psi. The sheath gas was kept at a flow rate of 10 L/min at a temperature of 350 °C. The voltages of the Fragmentor, Skimmer 1, and OctopoleRFPeak were 135, 50 and 500 V, respectively. The mass data were collected between *m/z* 80 and 1700 at the acquisition rate of 2 scans per second. Two reference masses at *m/z* 121.0509 (protonated molecular ion of C_5_H_4_N_4_) and *m/z* 922.0098 (protonated molecular ion of C_18_H_18_O_6_N_3_P_3_F_24_) for positive mode, and *m/z* 119.0363 (deprotonated molecular ion of C_5_H_4_N_4_) and *m/z* 966.0007 (formate adduct of C_18_H_18_O_6_N_3_P_3_F_24_) for negative mode were used as constant mass correction during LC-MS run. Product ion scanning experiments were conducted using ultra-high purity N_2_ as collision energy with same parameters set in MS acquisition, the collision energy (CE) was set at 10, 20 or 40 eV to generate the best quality of MS/MS spectra for the putative identification and structural elucidation of the significant metabolites.

### 4.5. Data Processing and Statistical Analysis

Multivariate and univariate analysis was performed to identify molecular features that discriminate melioidosis patients from patients with other bacteremia patients and controls as described previously [37]. Multivariate analysis was performed on a total of 76 LC-MS data of plasma samples from three groups (22, 24 and 30 samples from melioidosis patients, bacteremia with other bacteremia and controls without active infection respectively). The raw LC-MS data were converted into mzData format using Agilent MassHunter Qualitative Analysis software (version B.05.00, Agilent Technologies, USA) and subsequently processed using open-source XCMS package [39] operating in R [56], which adopted different peak detection and alignment as well as data filtering with centWave algorithms. Data was further processed with normalization, scaling, filtering and statistical analysis using MetaboAnalyst 3.0 [40]. The data were mean-centered, square root scaled and normalized such that the sum of squares for each chromatogram equaled on for statistical analysis [57]. Insignificant features between melioidosis patients and patients with bacteremia or controls were filtered out using both uni- and multivariate analyses. For multivariate analysis, PCA was performed for unsupervised analysis on all LC-MS features using MetaboAnalyst 3.0.

For univariate analysis, statistical significance of features was determined among melioidosis patients, patients with other bacteremia and controls using one-way ANOVA with Tukey’s *post-hoc* test. *p* < 0.05 was considered to be statistically significant. Significant features with FC >1.5 by volcano plots and *p* < 0.05 by Student’s *t*-test between melioidosis patients and patients with bacteremia, and between melioidosis patients and controls were identified. Common significant features were subject to univariate ROC analysis using web-based ROCCET [58]. The classical ROC curve analysis was performed and AUC was calculated by Monte Carlo Cross Validation (MCCV) using sub-sampling. In addition, the optimal cutoffs for the given metabolite were computed to obtain the sensitivity, specificity, and confidence intervals at different cut-offs for the evaluation of the recognition and prediction ability with respect to each variable. Significant features with AUC ≥0.8 obtained from either comparison between melioidosis patients and patients with other bacteremia, or between melioidosis patients and controls were identified. Box-whisker plots were generated and *p* values were calculated by the Student’s *t*-test using Analyse-it software (Analyse-it Software, Leeds, UK). Multivariate ROC curves were further generated using ROCCET. The procedures were repeated multiple times to calculate the performance and confidence interval of the model using support vector machines (SVM). The predicted class probabilities for each sample were evaluated with the best classifier (based on AUC) with confusion matrix.

### 4.6. Metabolite Identification

Features with significant differences were selected for product ion scanning (PIS) experiments. MS/MS spectra for the potential biomarkers and commercially available reference standards, including decanoylcarnitine, decanoylcarnitine, l-octanoylcarnitine, LysoPE(16:0/0:0), LysoPE(18:0/0:0) and PC(16:0/16:0), were processed using Agilent MassHunter Qualitative Analysis software (version B.05.00, Agilent Technologies, USA) to generate potential molecular formula based on the accurate mass and isotopic pattern recognitions of parent and fragment ions. All putative identities were confirmed by matching with entries in the METLIN database [59], Human Metabolome Database (HMDB) [60], MassBank [61], LipidMaps [62], KEGG (Kyoto Encyclopedia of Genes and Genomes) database [63] using exact molecular weights, nitrogen rule or MS/MS fragmentation pattern data and literature search. Efforts were made to distinguish metabolites from the other isobaric compounds whenever possible by its elution order and virtue of difference in fragmentation pattern corresponding to its structural characteristics. Putative identities of six biomarkers were confirmed by comparing their chromatographic RT and MS/MS spectra with those obtained from commercially available standards of decanoylcarnitine, decanoylcarnitine, l-octanoylcarnitine, LysoPE(16:0/0:0), LysoPE(18:0/0:0) and PC(16:0/16:0). The collision energy was set at 20 eV for generating the MS/MS spectra.

## 5. Conclusions

In this study, we compared the metabolome profiles of plasma of patients with melioidosis to those of patients with other bacteremia and controls without active infections. Twelve significant metabolites with significantly higher levels were identified in melioidosis patients than bacteremia patients and controls. These 12 metabolites, including l-octanoylcarnitine, decanoylcarnitine, dodecanoylcarnitine, LysoPE(16:0/0:0), LysoPE(18:0/0:0), PC(16:0/16:0), LysoPE(0:0/18:0), l-hexanoylcarnitine, SM(d16:1/16:0), 2-decenoylcarnitine, SM(d18:2/16:0) and *trans*-2-dodecenoylcarnitine, are involved in various pathways for energy and lipid metabolism. The present study demonstrates the potential of metabolomics in identifying novel biomarkers in studying infectious diseases. Further studies may reveal the potential of these metabolites as diagnostics biomarkers for melioidosis and their possible role in the pathogenesis and host response in melioidosis.

## Figures and Tables

**Table 1:** Plasma metabolites with higher levels in melioidosis patients compared to bacterimia patients and controls.

Compound	Experimental Mass, *m*/*z*	Ion	Retention Time (min)	MS/MS Fragment Masses	Elemental Composition	Metabolite Class
l-Hexanoylcarnitine	260.1842	[M + H]^+^	4.47	60.0808, 85.0285, 99.0803, 144.1018, 201.1117	C_13_H_25_NO_4_	acylcarnitine
l-Octanoylcarnitine	288.2157	[M + H]^+^	6.78	60.0805, 85.0283, 127.1110, 144.1019, 229.1438	C_15_H_29_NO_4_	acylcarnitine
2-Decenoylcarnitine	314.2326	[M + H]^+^	7.84	60.0806, 85.0281, 144.1015, 153.1257, 255.1591	C_17_H_31_NO_4_	acylcarnitine
Decanoylcarnitine	316.2476	[M + H]^+^	8.62	60.0806, 85.0284, 144.1017, 155.1424, 257.1748	C_17_H_33_NO_4_	acylcarnitine
*Trans*-2-dodecenoylcarnitine	342.2636	[M + H]^+^	9.38	60.0807, 85.0283, 144.1019, 181.1584, 283.1880	C_19_H_35_NO_4_	acylcarnitine
Dodecanoylcarnitine	344.2775	[M + H]^+^	10.20	60.0806, 85.0284, 144.1008, 183.1735, 285.2088	C_19_H_37_NO_4_	acylcarnitine
LysoPE(16:0/0:0)	454.2934	[M + H]^+^	14.64	44.0496, 62.0598, 216.0642, 239.2361, 257.2530, 313.2740, 393.2423, 436.2774	C_21_H_44_NO_7_P	lysophosphatidylethanolamine
LysoPE(0:0/18:0)	482.3244	[M + H]^+^	16.94	44.0494, 216.0628, 267.2644, 285.2747, 341.3060	C_23_H_48_NO_7_P	lysophosphatidylethanolamine
LysoPE(18:0/0:0)	482.3251	[M + H]^+^	17.61	44.0497, 62.0600, 216.0618, 267.2672, 285.2777, 341.3058, 421.2718, 464.3112	C_23_H_48_NO_7_P	lysophosphatidylethanolamine
SM(d16:1/16:0)	675.5444	[M + H]^+^	27.88	60.0808, 104.1072, 184.0735, 236.2355	C_37_H_75_N_2_O_6_P	sphingomyelins
SM(d18:2/16:0)	701.5605	[M + H]^+^	28.64	60.0802, 104.1068, 184.0736, 262.2575, 683.5484	C_39_H_77_N_2_O_6_P	sphingomyelins
PC(16:0/16:0)	734.5616	[M + H]^+^	30.58	60.0801, 104.1060, 184.0727, 478.3251, 496.3353	C_40_H_80_NO_8_P	phosphatidylcholine

**Table 2:** The area-under-receiver operating characteristic curve (AUC), sensitivity and specificity for receiver operating characteristic (ROC) curves calculated at optimal cutoff as well as *p*-value and Fold-change for the twelve significant metabolites.

Significant Metabolites	Melioidosis *vs.* Bacteremia						Melioidosis *vs.* Control without Active Infections					
	AUC ^a^	95% CI ^b^	Sensitivity (%)	Specificity (%)	*p*-Value ^c^	Fold-Change	AUC ^a^	95% CI ^b^	Sensitivity (%)	Specificity (%)	*p*-Value ^c^	Fold-Change
l-Hexanoylcarnitine	0.665	0.497–0.805	81.8	50.0	1.12 × 10^−2^	2.32 ↑^d^	0.849	0.728–0.959	83.3	81.8	1.10 × 10^−3^	3.33 ↑^d^
l-Octanoylcarnitine	0.856	0.710–0.964	86.4	79.2	6.34 × 10^−4^	3.49 ↑	0.827	0.693–0.942	83.3	86.4	2.90 × 10^−3^	2.76 ↑
2-Decenoylcarnitine	0.839	0.700–0.937	77.3	79.2	4.77 × 10^−3^	3.63 ↑	0.829	0.682–0.952	90.0	81.8	1.90 × 10^−3^	3.56 ↑
Decanoylcarnitine	0.850	0.728–0.972	77.3	87.5	1.46 × 10^−4^	3.53 ↑	0.821	0.680–0.949	83.3	77.3	1.45 × 10^−3^	2.66 ↑
*Trans*-2-dodecenoylcarnitine	0.850	0.736–0.965	77.3	79.2	1.20 × 10^−5^	2.40 ↑	0.886	0.776–0.965	90.0	72.7	5.43 × 10^−8^	2.88 ↑
Dodecanoylcarnitine	0.822	0.700–0.935	72.7	91.7	3.10 × 10^−5^	2.46 ↑	0.741	0.583–0.877	76.7	72.7	9.60 × 10^−3^	1.66 ↑
LysoPE(16:0/0:0)	0.979	0.947–1.000	90.9	95.8	1.51 × 10^−10^	5.20 ↑	0.812	0.672–0.933	73.3	81.8	2.56 × 10^−6^	2.01 ↑
LysoPE(0:0/18:0)	0.994	0.982–1.000	95.5	100.0	6.08 × 10^−10^	7.51 ↑	0.819	0.658–0.926	90.0	77.3	1.96 × 10^−6^	2.23 ↑
LysoPE(18:0/0:0)	0.998	0.993–1.000	100.0	95.8	1.77 × 10^−11^	6.09 ↑	0.856	0.725–0.958	90.0	77.3	1.41 × 10^−7^	2.16 ↑
SM(d16:1/16:0)	0.968	0.927–1.000	90.9	91.7	7.19 × 10^−10^	3.41 ↑	0.884	0.783–0.983	83.3	77.3	2.67 × 10^−8^	2.16 ↑
SM(d18:2/16:0)	0.998	0.993–1.000	100.0	91.7	1.88 × 10^−12^	3.32 ↑	1.000	1.000–1.000	96.7	100.0	1.28 × 10^−13^	2.65 ↑
PC(16:0/16:0)	0.835	0.695–0.976	77.3	87.5	7.01 × 10^−5^	9.64 ↑	0.870	0.724–0.989	93.3	81.8	2.77 × 10^−6^	21.72 ↑

^a^ AUC = area-under-receiver operating characteristic curve; ^b^ CI = confidence interval; ^c^ All *p*-values were calculated using Student’s *t*-test; ^d^↑ = Higher level comparing melioidosis to the respective groups.

## Abbreviations

ACS	American Chemical Society
ANOVA	analysis of variance
ATII	alveolar type II pneumocytes
AUC	area-under-receiver operating characteristic curve
CE	collision energy
DPCC	dipalmitoylphosphatidylcholine
FA	fatty acid
FC	fold-change
IL-6	interleukin-6
IL-8	interleukin-8
LysoPE	lysophosphatidylethanolamine
MCCV	Monte Carlo Cross Validation
PC	phosphatidylcholine
PCA	principal component analysis
PIS	product ion scanning
PLA2	phospholipase A2
PlcH	phospholipase C
ROC	receiver operating characteristic curve
RT	retention time
SM	sphingomyelins
SIRS	systemic inflammatory response syndrome
SVM	support vector machines
TCA	tricarboxylic acid
TNF-α	Tumor necrosis factor-α
UHPLC-ESI-QTOFMS	ultra-high-performance liquid chromatography-electrospray ionization-quadruple time-of-flight-mass spectrometry
VIP	variable importance in the projection
